# Genomic Insights into Achromobacter mucicolens *IA* Antibiotic Resistance

**DOI:** 10.1128/spectrum.01916-21

**Published:** 2022-04-04

**Authors:** Sura Ali Al-Asadi, Rusul Emaduldeen S. Al-Kahachi, Wifaq M. Ali Alwattar, Jamila Bootwala, Majeed Arsheed Sabbah

**Affiliations:** a Health and Medical Technology College Baghdad, Middle Technical University, Baghdad, Iraq; b Iraqi Ministry of Higher Education and Scientific Research, Deputy of Scholarships and Cultural Relationship, Baghdad, Iraq; c Unit of Clinical and Communicable Diseases, College of Medicine, Baghdad University, Baghdad, Iraq; d Genejenie, Genomics Department, Mumbai, India; e Forensic DNA Center, Al-Nahrain University, Baghdad, Iraq; USDA - San Joaquin Valley Agricultural Sciences Center

**Keywords:** microbial antibiotic resistance, bioinformatics, epidemiology, microbial pathogenesis, antibiotic resistance, antibiotic susceptibility, microbial pathogen

## Abstract

Achromobacter denitrificans is an environmental opportunistic pathogen that is infecting a large number of immunocompromised patients. A more recently identified strain from the historical collection of strains of Achromobacter denitrificans is Achromobacter mucicolens. In hosts with a variety of underlying diseases, *Achromobacter* spp. can induce a wide spectrum of disorders. Because of the bacterium’s intrinsic genetic constitution and resistance gained over time, antibiotics are challenged to handle *A. mucicolens.* Due to the fact that *A. mucicolens* is rare and its taxonomy is not completely understood, it is difficult to define clinical symptoms, acquisition risk factors, and thus the best therapeutic course of action. To help comprehend this intrinsic and acquired resistance, we analyzed the entire genome of the *A. mucicolens IA* strain and utilized bioinformatics methods to estimate the strain's probable drug resistance profile. In our study, we have isolated and cultured a clinically important *A. mucicolens* strain and subjected it to antimicrobial susceptibility tests against antibiotics in the Vitek 2 testing system. The strain’s genome sequence as well as an investigation of 27 of its phenotypic traits provides important information regarding this pathogen. The genome of this *A. mucicolens IA* strain possesses a number of antibiotic resistance genes that code for efflux pump systems and other antibiotic-regulating as well as -modifying enzymes. Our research analysis predicted genes involved in drug resistance, including genes for efflux pump systems, antibiotic efflux, antibiotic inactivation, and antibiotic target alteration. *In vitro* studies validated the genomic evidence for its ability to exhibit resistance against a wide range of antibiotics. Our investigation paves the way for more research on understanding the functioning of the key discovered genes that contribute toward the pathogenicity of *A. mucicolens* and hence gives new information and treatment options for this emerging pathogen.

**IMPORTANCE** *Achromobacter* species are well-known opportunistic human pathogens that can be found in water and soil and most commonly in hospital settings. They thrive in immunocompromised individuals, producing sporadic cases of pneumonia, septicemia, peritonitis, urinary tract infections, and other illnesses. *Achromobacter* strains are inherently resistant to a wide spectrum of antibiotics, making them difficult to treat promptly. The strain under study, *A. mucicolens*, was notably resistant to various antibiotics, and the infection could be controlled only after several rounds of prescription medications at different doses. This consumed a lot of time and put the already immunosuppressed leukemic patient through a great ordeal. The study aimed to raise awareness about the importance of the *Achromobacter* bacterium’s lethality, and doctors should evaluate the bacterium’s potential for resistance before prescribing antibiotics. Sanitation and other precautions should also be implemented in hospitals and other public places.

## INTRODUCTION

Antibiotic-resistant bacteria are rapidly mutating, and their massive spread is being portrayed as one of the most adverse health issues in the world (1). This rising resistance to numerous antibiotics requires a deeper understanding of the causes and hot spots that contribute to its emergence and spread. So far, antibiotic resistance mechanisms have evolved in opportunistic and pathogenic bacteria through changes in already existing genes on the bacterial chromosome that are selected for by environmental factors (2, 3). The mutations that occur in the chromosome are responsible for the bacteria’s reduced antibiotic affinity. Moreover, a number of resistance mechanisms, such as efflux pumps and chromosomal AmpC β-lactamases, have their expression regulated on a basal level, resulting in decreased drug susceptibility, naturally. Overexpression and a high level of antibiotic resistance are the results of mutations that affect genomic structures and processes (4, 5). Pressure to develop the resilience of antibiotics, on the other hand, expedites the integration of antibiotic resistance genes from donor species through lateral transfer (6). Horizontal gene transfer events are responsible for the procurement of heterologous resistance genes from other bacterial species. As a result, hospitals, farms, agriculture, aquacultures, the human community, and other places operate as reactors in which increased antibiotic use promotes the emergence of resistant bacteria and the transfer of genes. In addition, low-cost medications, preventative medicine utilizing broad-spectrum antibiotics, and the abuse of these drugs all have a role in the rise of drug-resistant bacteria (7).

The *Achromobacter* species are Gram-negative, nonfermenting rods that live in the human intestine and have limited inherent pathogenic potential. There are now 19 officially recognized species in the *Achromobacter* genus, with the majority of them having been discovered in the most recent decade (8). Fifteen species to date have been isolated from clinical specimens, including A. xylosoxidans, A. denitrificans, A. ruhlandii, and A. piechaudii (9); A. animicus, A. mucicolens, and A. pulmonis (10); A. insolitus and A. spanius (11); A. deleyi (12); A. aegrifaciens, A. insuavis, A. anxifer, and A. dolens (13); and A. marplatensis (14). Very recently, A. xylosoxidans has been subdivided into two subspecies, Achromobacter xylosoxidans subsp. *denitrificans* and Achromobacter xylosoxidans subsp. *xylosoxidans* (15). Among these, *A. denitrificans* is commonly found to inhabit aquatic sources and also the human gut. It can cause nosocomial and community-acquired infections, but in immunocompromised persons, invasive infections produced by *A. denitrificans* can be fatal. The majority of infections occur during hospitalization, with primary simple bacteremia, pneumonia, and catheter-associated infections being the most prevalent (16). The clinically most important species belonging to the genus *Achromobacter* have been regularly isolated from human samples obtained in various nosocomial illnesses related to the infusion of contaminated fluids, humidifiers, and incubators. Immunodeficiency, HIV infection, cancer, cystic fibrosis, and prolonged hospitalization are all risk factors for infection. Asymptomatic infections include clinical cases such as natural-valve or prosthetic valve endocarditis, pneumonia, peritonitis, meningitis, conjunctivitis, osteomyelitis, prosthesis infections, and intra-abdominal abscess, while symptomatic infections include pneumonia, peritonitis, meningitis, conjunctivitis, osteomyelitis, prosthesis infections, and intra-abdominal abscess (17–19).

A historical collection of microorganisms designated *A. denitrificans* was studied in terms of phenotypic and genotypic traits. According to sequence analysis of a 765-bp *nrdA* gene fragment, eight of the bacteria belonged to the newly described *A. aegrifaciens*, *A. mucicolens*, and *A. insolitus*, while one strain belonged to A. xylosoxidans (12).

Understanding the epidemiology of any infection can happen only with a proper investigation of the causative organism at a genetic level. In this study, we used bioinformatics tools and software to analyze the entire genome sequence of the *A. mucicolens IA* strain RefSeq NZ_CP082965.1 to provide a comprehensive description of the various drug class families to which our strain is resistant.

## RESULTS

**FIG 1:**
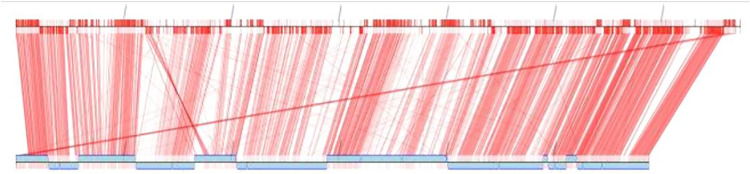
Mapping of the assembled genomic sequence of the Achromobacter mucicolens *IA* strain using CONTIGuator 2.7.4.

**FIG 2:**
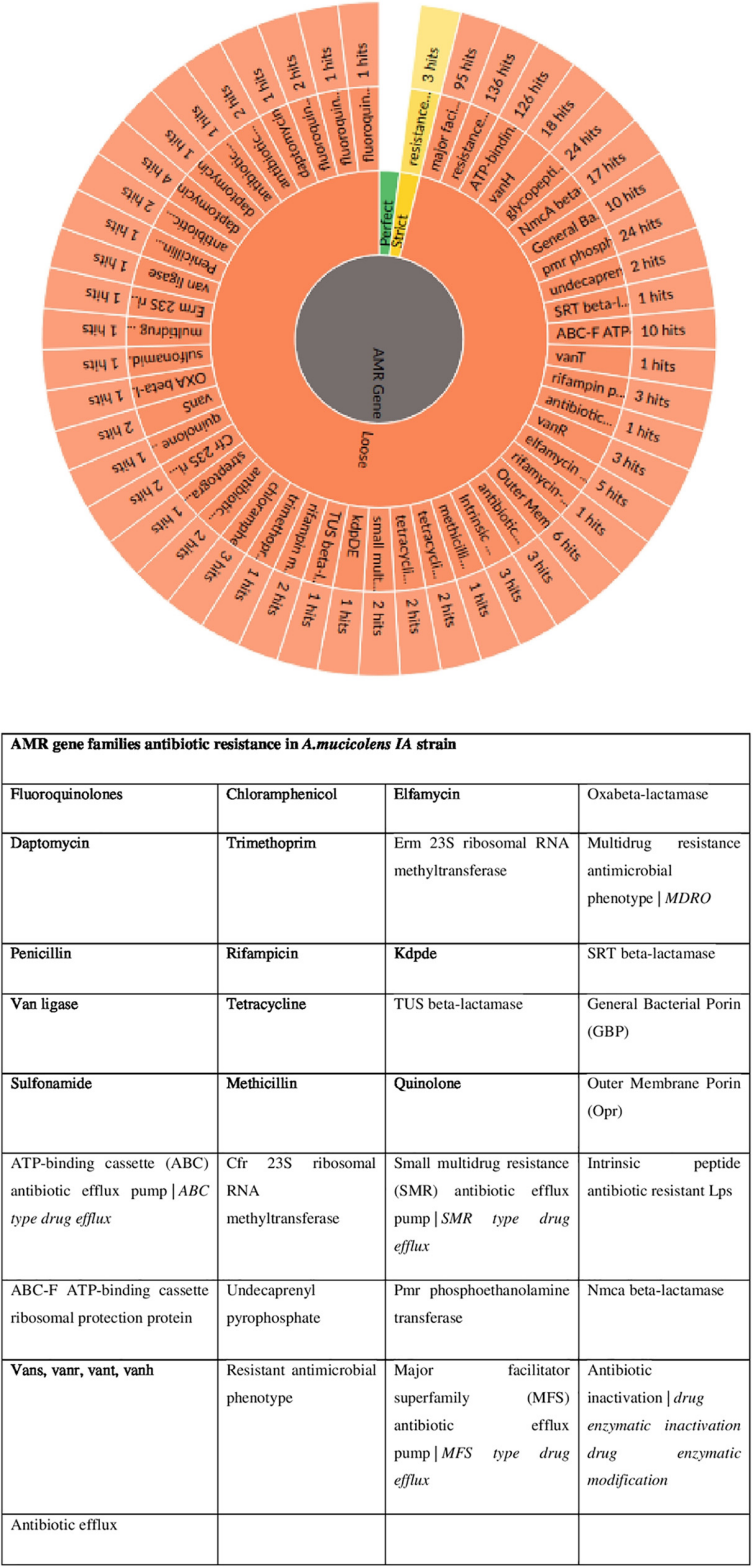
Predicted resistome and CARD-generated visualizations for the AMR gene family corresponding to drug resistance in the *A. mucicolens IA* strain. (Top) CARD antimicrobial resistance (AMR) detection models include a reference sequence, a curated BLAST (P/N) bit score cutoff, and, if applicable, mutations known to predict AMR. (Bottom) User-submitted queries are analyzed using detection models which generate an annotation organized by the Perfect, Strict, and Loose (if selected) paradigm.

**FIG 3:**
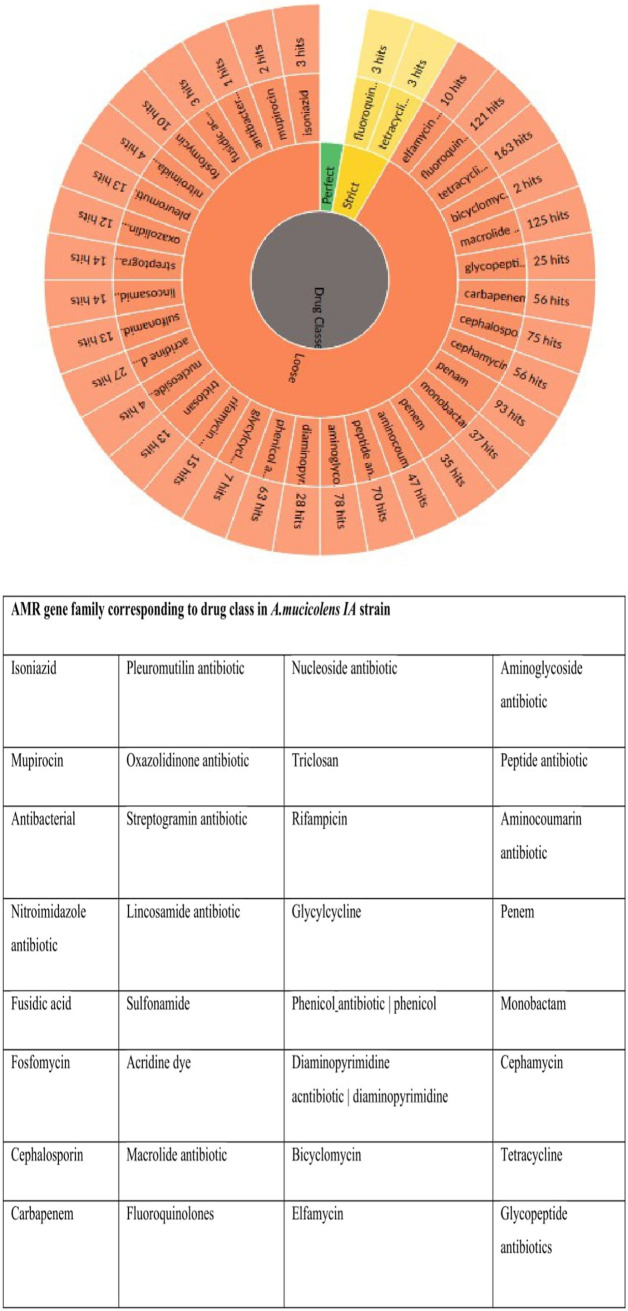
CARD-generated visualizations for the AMR gene family corresponding to drug class in the *A. mucicolens IA* strain. User-submitted queries are analyzed using detection models which generate an annotation organized by the Perfect, Strict, and Loose (if selected) paradigm.

**FIG 4:**
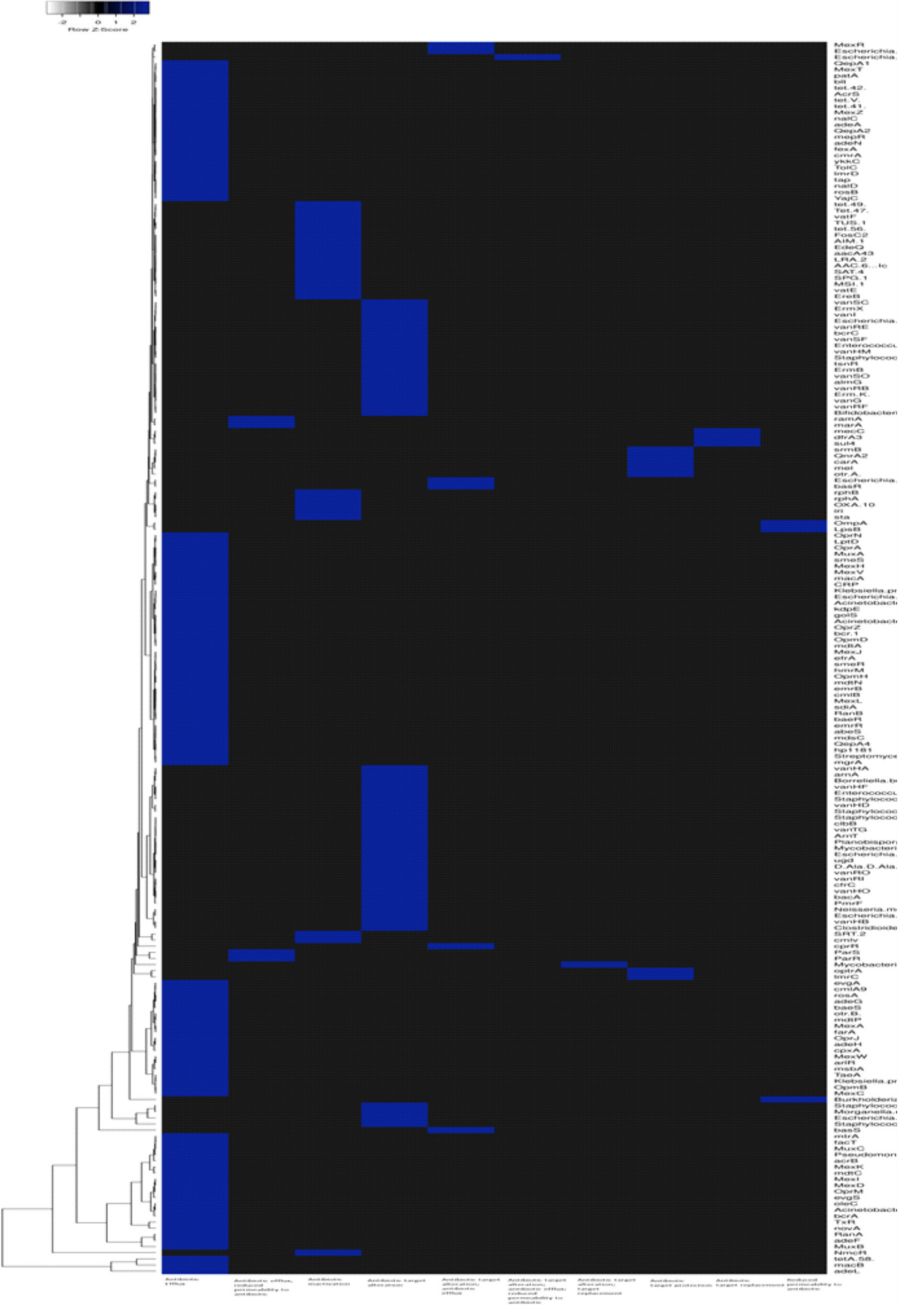
Heatmap showing antibiotic resistance genes present in the Achromobacter mucicolens *IA* strain obtained based on the Euclidean algorithm. The information on the *x* axis shows the resistance mechanism found in the sample in accordance with the AMR genes found using CARD.

The identification of our strain was primarily conducted using the biochemical methods of the Vitek 2 system (bioMérieux), shown in Table 1, and the results reported the *A. mucicolens* strain as *A. denitrificans.* However, whole-genome sequencing and sequence analysis of the *nrdA* 765-bp sequence obtained from PubMLST clarified and confirmed the strain as *A. mucicolens.* The MICs of several antibiotics reported for the *A. mucicolens* isolate using an automated microbiology identification system were used in antimicrobial susceptibility testing by the Vitek 2 system as shown in Table 2. Mapping of the assembled genomic sequence of the *A. mucicolens IA* strain was performed using CONTIGuator 2.7.4 (Fig. 1). The assembly was found to have 23 contigs, 5,885,078 bp. In accordance with the Comprehensive Antibiotic Resistance Database (CARD), the antimicrobial resistance (AMR) gene families of *A. denitrificans* were used to identify the drug resistance in *A. mucicolens* (Fig. 2). Using the same database, the genes which show resistance to different drug classes were classified (Fig. 3), which corresponds to antimicrobial susceptibility tests conducted. The same was represented in the form of a heatmap that was built based on the Euclidean algorithm (Fig. 4). Approximately 208 genes were predicted as shown in the heatmap that are involved as drug resistance-related enzymes and genes of antibiotic efflux pump systems (see supplementary file 1 in the supplemental material). Out of 208 predicted genes, 111 genes are involved in the antibiotic efflux system with gene functions such as ATP-binding cassette (ABC) antibiotic efflux pump, resistance-nodulation-cell division (RND) antibiotic efflux pump, major facilitator superfamily (MFS) antibiotic efflux pump, 25 genes in antibiotic inactivation, and 68 genes in antibiotic target alteration. Eight genes are involved in antibiotic efflux and reduced permeability to antibiotics.

**TABLE 1:** Biochemical studies done on the isolated strain of Achromobacter mucicolens using the Vitek 2 system (bioMérieux)^*a*^

Test no.	Biochemical test	Result
2	APPA	−
3	ADO	−
4	PyrA	+
5	IARL	−
7	dCEL	−
9	BGAL	−
10	H2S	−
11	BNAG	−
12	AGLTp	+
13	dGLU	−
14	GGT	−
15	OFF	−
17	BGLU	−
18	dMAL	−
19	dMAN	−
20	dMNE	−
21	BXYL	−
22	BAlap	−
23	ProA	+
26	LIP	−
27	PLE	−
29	TyrA	+
31	URE	−
32	dSOR	−
33	SAC	−
34	dTAG	−
35	dTRE	−
36	CIT	+
37	MNT	−
39	5KG	−
40	ILATk	+
41	AGLU	−
42	SUCT	+
43	NAGA	−
44	AGAL	−
45	PHOS	+
46	GlyA	−
47	ODC	−
48	LDC	−
53	IHISa	+
56	CMT	−
57	BGUR	−
58	O129R	−
59	GGAA	−
61	IMLTa	−
62	ELLM	−
64	ILATa	−

a The table shows various biochemical methods for measuring carbon source utilization, enzymatic activities, and resistance. It typically identifies the quality control organisms as one choice or within low discrimination or slashline identification. +, 95% to 100% positive; v, variable; 6% to 94% positive; −, 0% to 5% positive.

**TABLE 2:** Antimicrobial susceptibility of isolated organism Achromobacter mucicolens against standard drugs using Vitek 2 system (bioMérieux)^*a*^

Antimicrobial	MIC (μg/ml)	Interpretation
Piperacillin-tazobactam	≥128	R
Cefazolin	≥64	R
Ceftazidime	≥64	R
Ceftriaxone	≥64	R
Cefepime	≥64	R
Imipenem	2	S
Amikacin	16	S
Gentamicin	8	I
Ciprofloxacin	2	I
Levofloxacin	≥8	R
Tigecycline	≤0.5	S
Trimethoprim-sulfamethoxazole	160	R

a MIC represents MIC values with different tested drugs. Interpretation of antibiogram: S, sensitive—active substance normally effective against microorganisms at the recommended dosage; I, intermediate—active substance may be effective against microorganisms at higher than the recommended dose; R, resistant—an active substance not effective against microorganism in either recommended or higher dosage due to resistance mechanism.

### Phage detection.

**FIG 5:**
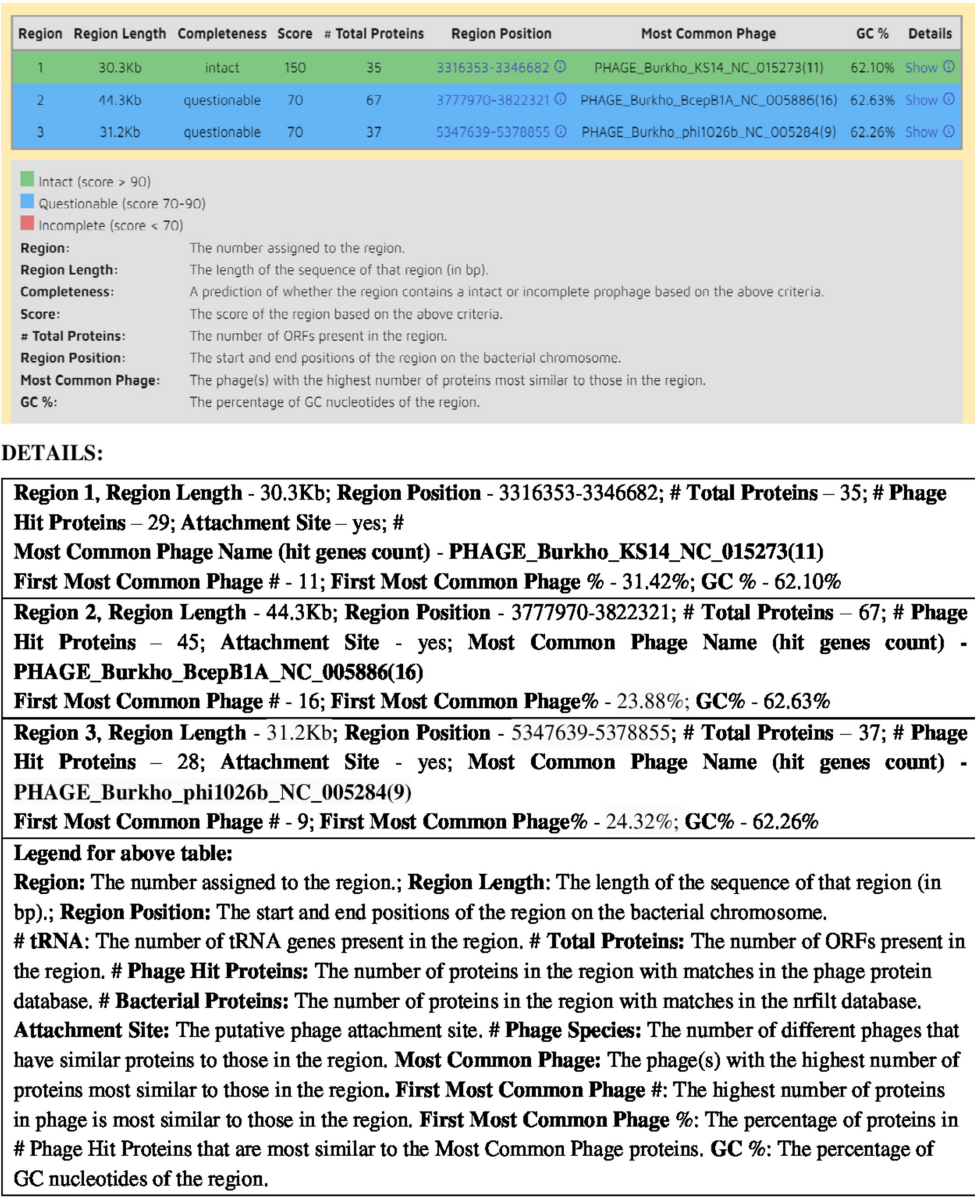
Results from PHASTER, identifying and annotating the most common phage sequences within the *IA* strain with the respective scoring confidence with region length and region position, the details of which are listed in the table below for the rapid identification and annotation of prophage sequences within bacterial genomes and plasmids.

**FIG 6:**
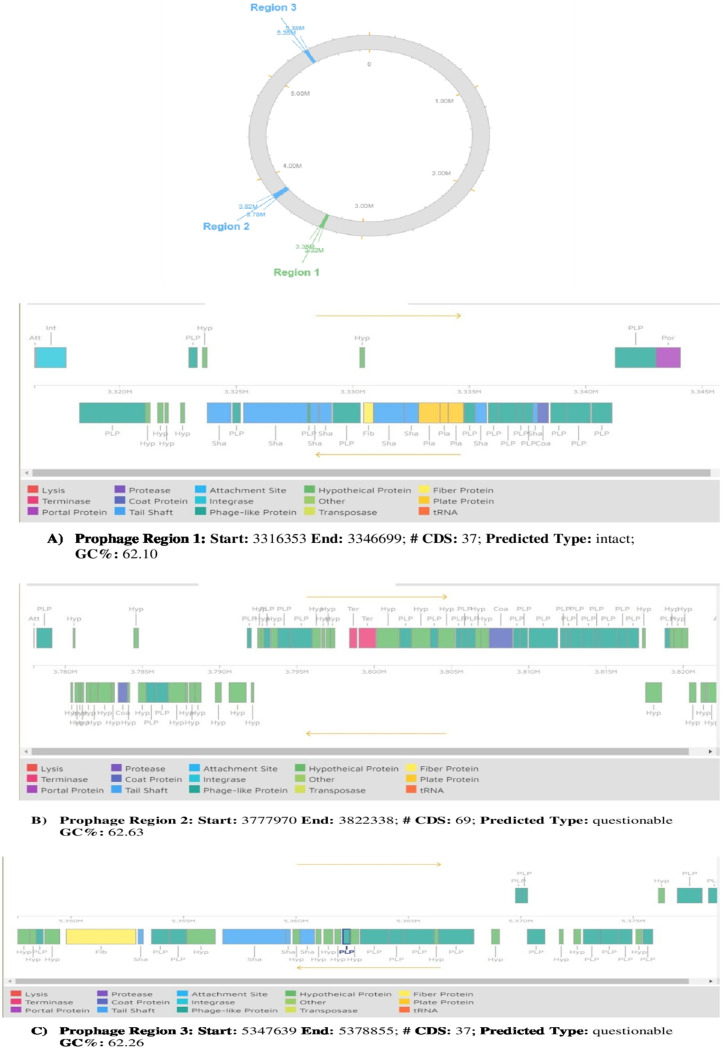
Circular map representation of the locations of the three regions, region 1, region 2, and region 3, on the whole genome of the *A. mucicolens IA* strain. Panels A to C show the detailed phage protein-encoding sequence of region 1, region 2, and region 3 as depicted on top.

Prophages are one of the most important sources of genetic diversity and strain variation related to bacterial pathogenicity. For identification and annotation of phage sequences, which are major horizontal gene transfer agents included in the mobilome within bacterial genomes, the tool Phaster was used. Results in Fig. 5 and 6 show that our *IA* strain is predicted to have 3 phage sequences, named region 1, region 2, and region 3. Region1 possesses proteins that are most similar to the highest number of proteins found in PHAGE_Burkho_KS14_NC_015273 (11 hit gene count), 31.42% of proteins with the highest similarity to the most common phage proteins. Region2 has proteins with the maximum similarity to the highest number of proteins found in PHAGE_Burkho_BcepB1A_NC_005886 (16 hit gene count), 23.88% of proteins that are most similar to the most common phage proteins. Region3 possesses proteins with the maximum similarity to the highest number of proteins found in PHAGE_Burkho_phi1026b_NC_005284 (9 hit gene count), 24.32% of proteins that are highly similar to the most common phage proteins.

### GIs.

**FIG 7:**
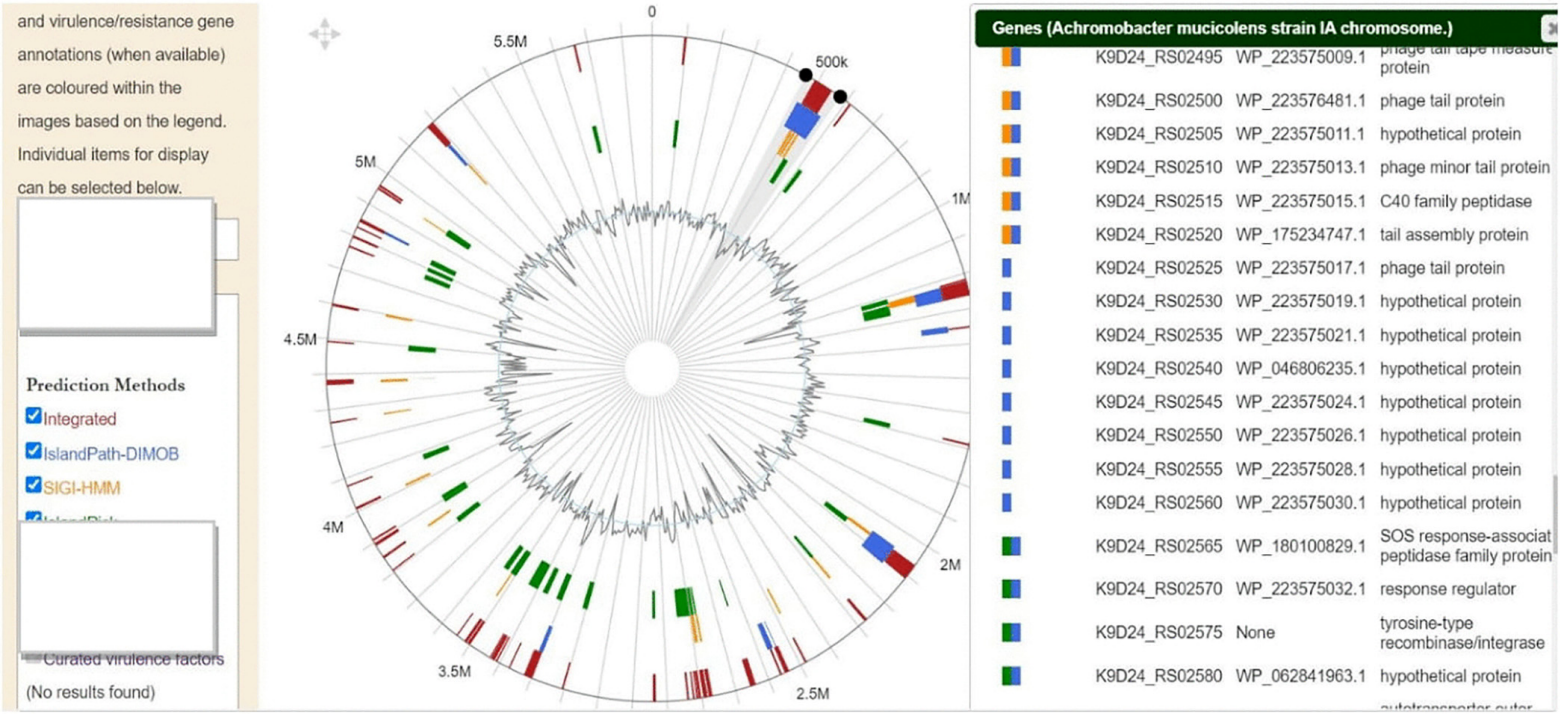
Predictions of genomic islands in the Achromobacter mucicolens *IA* strain genome (accession number NZ_CP082965.1). Circular (left) and linear (right) visualizations of predicted genomic islands are shown, with blocks colored according to the prediction method, IslandPick (green), IslandPath-DIMOB (blue), and SIGI-HMM (orange) as well as the integrated results (dark red).

A total of 6,51,854 bp in length (11.07% of the whole genome) was determined as genomic islands (GIs) in the *A. mucicolens IA* strain assembly. All GIs were substantially dissimilar and separate from each other (Fig. 7). Each of the GIs encoded both putative and functional proteins. The GIs’ regions showed the presence of genes usually found on GIs that are recognized as site-specific integrase protein, phage structural proteins, tyrosine-type recombinase/integrase, and viral recombinase family protein. Further analysis of genomic islands predicted drug resistance genes found for arsenical resistance protein, multidrug efflux RND transporter protein, and class D β-lactamases, located in a putative genomic island in this *A. mucicolens* strain, indicating that these genes are probably acquired through horizontal gene transfer (see supplementary file 2).

### TRs.

A total of 407 tandem repeats (TRs) were identified in the *A. mucicolens IA* strain assembly with period sizes ranging from 3 to 393 bp. The total TR length and percentage of genome coverage for period size were 11,511 bp and 0.195%, respectively. Many of the TRs identified in *A. mucicolens* were minisatellites (10 to 100 bp), with 42% of all repeats located in protein-coding regions (see supplementary file 3).

### Secondary metabolite gene clusters.

**FIG 8:**
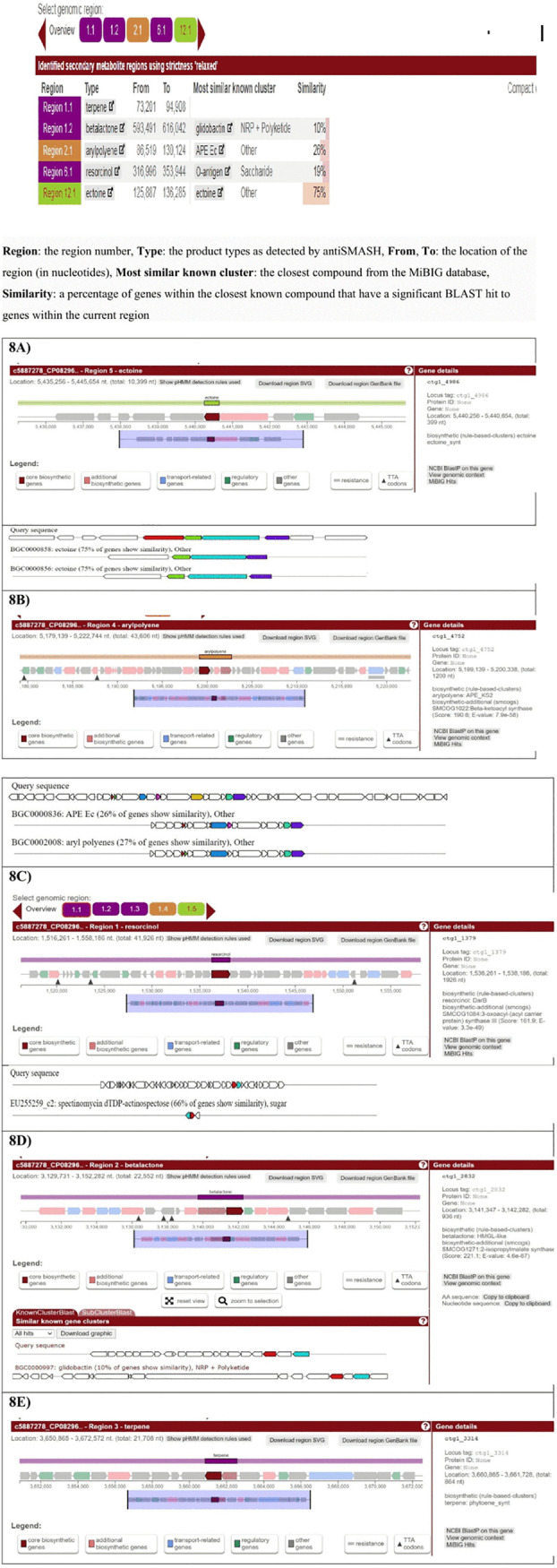
Results of antiSMASH 6.0 depicting secondary metabolite biosynthesis gene clusters in the *A. mucicolens IA* strain. Panels A to E are each laid out as follows. In the upper panel, “Gene cluster description,” information is given about each gene cluster that was detected. In the upper line, the biosynthetic type and location of the gene cluster are displayed. Underneath this title line, all genes present in a detected gene cluster are outlined. The borders of the gene clusters have been estimated using different chosen cutoffs specified per gene cluster type. Genes are color coded by predicted function. Putative biosynthetic genes are colored red, transport-related genes are colored blue, and regulation-related genes are colored green.

A region in the antiSMASH corresponds to the gene cluster annotation, and the similarity shows the percent similarity of genes in the nearest known compound that has a substantial BLAST hit against genes in the present location. The *A. mucicolens IA* strain assembly contained five secondary metabolite regions (Fig. 8). The region showed maximum similarity of 75% to a most similar known gene cluster of ectoine having its product as ectoine. The clusters encompassed a total of 10,398 bp (genomic positions 125,887 to 136,285 bp) with a core biosynthetic gene producing ectoine synthase. The region with 26% similarity to the most similar known gene cluster of APE Ec produces arylpolyene. The clusters spanned a total of 43,605 bp (genomic positions 86,519 to 130,124 bp) with a core biosynthetic gene producing APE_KS1 and APE_KS2, beta-ketoacyl synthase. The region with 66% similarity to the most similar known gene cluster of spectinomycin dTDP-actinospectose produces resorcinol. The clusters encompassed a total of 36,998 bp (genomic positions 316,996 to 353,994 bp) with core biosynthetic gene DarB, producing 3-oxoacyl-acyl carrier protein (ACP) synthase III. The region with 10% similarity to the most similar known cluster of glidobactin produces beta-lactone. The clusters encompassed a total of 22,551 bp (genomic positions 593,491 to 616,042 bp) with a core biosynthetic gene producing AMP-dependent synthetase and ligase and 2-isopropylmalate synthase. The last product is terpene, spanning a total of 21,707 bp (genomic positions 73,201 to 94,908 bp), with a core biosynthetic gene producing phytoene synthase (Fig. 8A to C).

### COG database.

**FIG 9:**
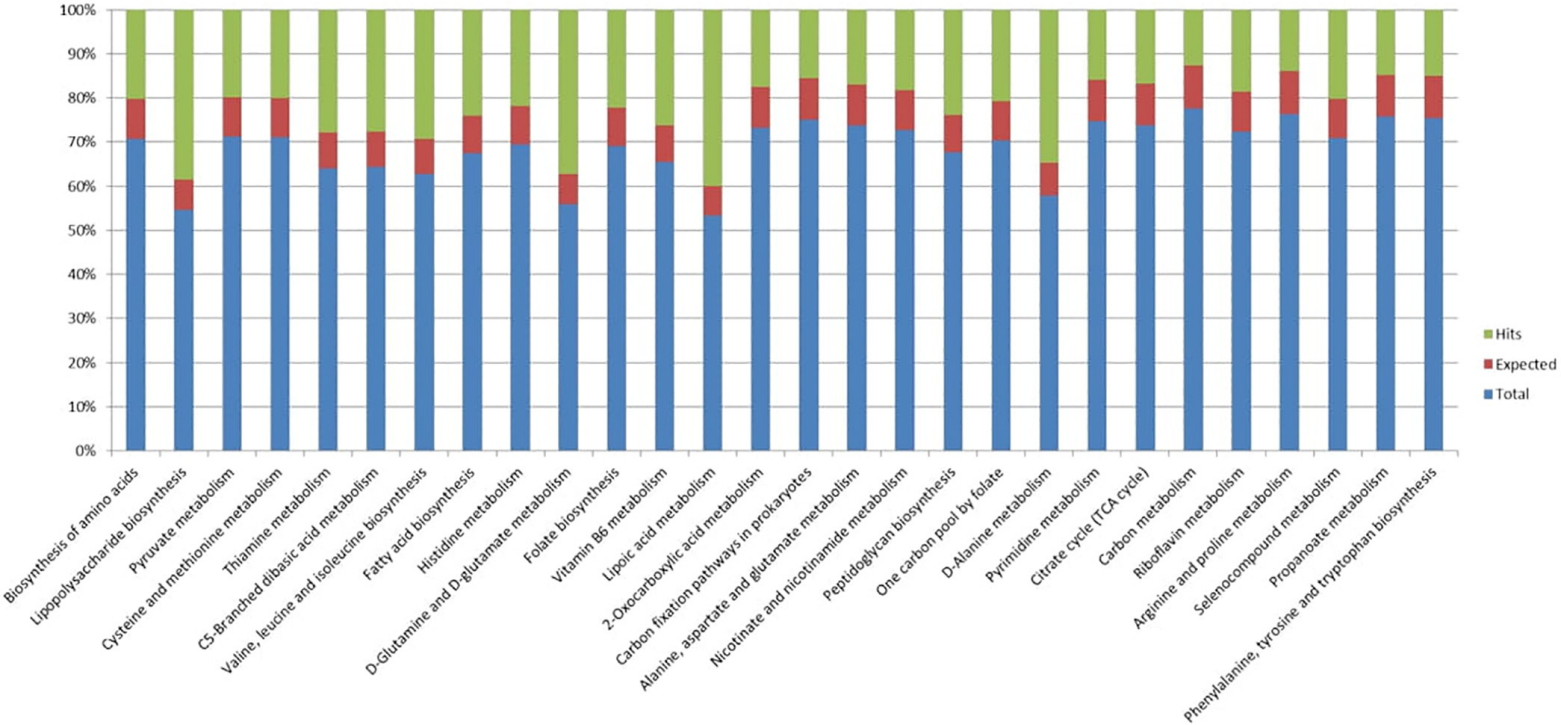
COG database bar chart representation showing the distribution of metabolic pathways of Clusters of Orthologous Groups (COGs) for predicted protein-coding genes for the *A. mucicolens IA* strain. Bars are color coded according to the genome (see key): green, hits observed in the sample; red, those expected to be present in the sample; blue, total number of pathways present in the database.

The Clusters of Orthologous Genes (COG) database was used to identify pathways encoded by the gene for a protein present in the *A. mucicolens IA* strain (Fig. 9). Up to 10 β-lactamase-encoding Antibiotic Resistance Ontology (ARO) genes belonging to the AMR gene family were predicted**—**these are AIM β-lactamase (AIM-1), MSI β-lactamase (MSI-1), NmcA β-lactamase (NmcR), TUS β-lactamase (TUS-1), SPG β-lactamase (SPG-1), OXA β-lactamase (OXA-10), subclass B3 LRA β-lactamase (LRA-2), SRT β-lactamase (SRT-2), and penicillin-binding protein mutations conferring resistance to β-lactam antibiotics (Neisseria meningitidis PBP2 conferring resistance to β-lactam) (see supplementary file 4). The output obtained represented pathway hits observed in the sample, those expected to be present in the sample, and the total number of pathways present in the database. The above said annotation and presence of genes were confirmed with RAST 2.0, which is generally used for archaeal and bacterial genomes (see supplementary file 5).

## DISCUSSION

In our study, we tested the *A. mucicolens IA* strain whole-genome assembly for identification using the biochemical methods in the Vitek 2 system (bioMérieux). The results identified our strain as *A. denitrificans*. However, whole-genome sequencing with Illumina (NovaSeq 6000) and analysis of the *nrdA* gene 765-bp sequence from PubMLST confirmed the bacterial strain to be *A. mucicolens.* Hence, as opposed to the Vitek 2 test identification accuracy findings of Ligozzi et al., 2002 (20), we suggest using whole-genome sequencing as the most accurate method to achieve the correct differentiation of the bacterial taxonomy for Gram-negative bacteria, especially from *Achromobacter* species.

The susceptibility of *A. mucicolens* to 12 antimicrobial agents involving MICs was tested: *A. mucicolens* showed resistance to piperacillin-tazobactam, cefazolin, ceftazidime, ceftriaxone, cefepime, and trimethoprim-sulfamethoxazole and sensitivity to drugs imipenem, amikacin, and tigecycline. Trimethoprim-sulfamethoxazole had the highest MIC. This research piqued interest in researching antibiotic resistance genes and mechanisms that may play a role in resistance in the *A. mucicolens IA* strain. Furthermore, the *IA* whole-genome assembly was subjected to CARD search, which predicted the numerous AMR gene families resistant to antibiotics and different drug classes. A heatmap of the *IA* strain gave us numerous genes involved in drug resistance-related enzymes and genes of antibiotic efflux pump systems. Extrusion of numerous foreign and endogenous chemicals is controlled by efflux systems, which are membrane-located pump proteins found in all eukaryotic and prokaryotic cells. Some microorganisms have intrinsic resistance to antibiotics, which is mediated by efflux pumps. Pumps also contribute to other resistance mechanisms by causing acquired resistance through overexpression. Pumps can also increase the pathogenicity of bacteria, either directly or indirectly (21), reinforcing the possibility that the genes responsible for this resistance are either internal to the *Achromobacter* species or acquired over time from other bacteria. To investigate further, we used tools like Phaster and IslandViewer 4 to annotate prophage sequences within bacterial genomes and genomic islands, respectively. Phages aid in the horizontal transmission of genetic material and support the spread of antibiotic resistance genes in bacteria. Bacteria can acquire new genetic material either from within, through internal genetic mutation, or from outside, through horizontal gene transfer (HGT). Bacteria may rapidly acquire complex new features thanks to HGT, which has been and continues to be a major driving force in bacterial evolution (21–27). Horizontal gene transfer aids the spread of antibiotic resistance by allowing genetic material to cross genera, increasing the risk of development of dangerous, antibiotic-resistant bacteria (https://www.lakeforest.edu/live/files/the-role-of-horizontal-gene-transfer-in-antibiotic.pdf) (28, 29). We detected three phage regions, region 1, region 2, and region 3, with region 1 having a high score of 150, meaning that it has acquired or transferred a whole intact phage region from another bacterium and has it in its genome. The protein-coding sequence for region 1 has several genes encoding phage structural proteins and enzyme integrase, required for integration of the viral DNA to the host DNA. Region 2 showed the presence of gene sequences for phage-like proteins and terminase enzyme, to initiate packaging of the viral genome and, also, translocation. Region 3 has the presence of genes responsible for phage-like proteins and tail shaft proteins. These phage genes are frequently found transferred through horizontal gene transfer by mobilome: prophages and transposons (https://www.ncbi.nlm.nih.gov/research/cog/cogcategory/X/) (29, 30).

A genomic island (GI) is a section of a genome with evidence of horizontal ancestry. A GI can play a big role in pathogenesis, and it can also assist an organism to fight antibiotics. The IslandPick tool of IslandViewer 4 identifies regions that are unique to only one genome by comparing genomes that are within a reasonable evolutionary distance (31). Moreover, the results from our analysis identified regions that contain genes frequently carried on GIs such as those for site-specific integrase protein, phage structural proteins, tyrosine-type recombinase/integrase, and viral recombinase family protein (30). The presence of genes involved in phage structure and function on the genomic island indicates that they were acquired from other bacteria, which also transmitted resistance genes. The conclusion of our results is similar to a study that was carried out to show that consecutive acquisition of resistance determinants may have resulted in the formation of the resistance gene cluster inside the SGI1 genomic cluster. Southern blot hybridization and PCR amplification assays were used to learn more about the presence and conservation of the SGI1 genomic island in the DT104 strains. To explore the existence of SGI1 and to study antibiotic resistance genes carried by integrons, multidrug-resistant Salmonella enterica serovar Typhimurium strains of various phage types of both human and animal origin were studied using PCR amplification and Southern blot hybridization. The two integrons InC and InD, situated within SGI1, were present in all DT104 strains with the ACSSpSuT resistance profile (32, 33).

The bioinformatic analysis of tandem repeats reported a total of 407 tandem repeats (TRs) in the *A. mucicolens IA* strain. Tandem repeats are a pattern that can be used to determine inherited characteristics in bacteria. Tandem repeats can also be present in genes that code for important biological activities like DNA replication (33, 34). The link between TRs and cell surface structures has been proposed as a way for populations to anticipate environmental changes and improve their survival probability (35). Further comparative analysis of these tandem repeat regions to other *Achromobacter* strains can determine the inheritance of resistance in these tandem repeat regions. Furthermore, bacteria also benefit from the fact that they can transiently shut down or alter the function of specific genes, which allows them to adapt to changing environments in brief evolutionary timespans without increasing total mutation rates (36). Therefore, tandem repeats can be useful in determining the intrinsic resistance of the *A. mucicolens IA* strain and its role in bacterial survival.

In order to compete against other bacteria, fungi, amoebae, plants, insects, and large animals, bacteria biosynthesize secondary metabolites, including antibiotics, as a competitive weapon to help clear the nearby surroundings of microorganisms. Depending on hidden Markov models of genes with distinct profiles for different types of gene clusters, antiSMASH 6.0 accurately identified the gene clusters encoding secondary metabolites in our *A. mucicolens IA* strain. A region in the antiSMASH corresponds to the gene cluster annotation, and the percent similarity of genes in the nearest known compound that has a significant BLAST hit against genes in the current location is displayed. AntiSMASH results showed our strain to possess a genetic region of a secondary metabolite biosynthetic gene cluster, possessing a similarity of 75% with ectoine compound, having a significant BLAST hit to genes within the current region. This is followed by arylpolyene compound having a genetic cluster region similarity of 26%, resorcinol compound having a genetic cluster region similarity of 66%, beta-lactone compound having a genetic cluster region similarity of 10%, and lastly, terpene compound. Our results are in accordance with the secondary metabolites that induce the expression of oxidative stress responses, analogous to the protective effects of sublethal doses of oxidants such as H_2_O_2_, which can train bacterial cells for antibiotic tolerance and resistance (29). Another study found that coculturing *Actinomycetes* with antibiotic-resistant bacteria may promote the development of new secondary metabolites that are effective against them (37).

The COG database outcome suggests that the gene distribution varies in different subsystems with the same kinds of organisms isolated from different sources. Also, if all of the genes in the genomes are merged into the subsystems, the set of genes displayed will change. Furthermore, the total number of genes included in the various categories of the subsystem previously reported is not identical to the number of genes existing in the genome (38). To further investigate the antibiotic resistance profile of the *A. mucicolens IA* strain, we found 10 β-lactamase-encoding genes among Antibiotic Resistance Ontology (ARO) genes belonging to the AMR gene family through the COG database and confirmed it with RAST 2.0. The β-lactamase gene is a narrow-spectrum gene that likely possesses a secondary role for the final β-lactam resistance profile of *Achromobacter* species. The synthesis of different β-lactamases, which hydrolyze the β-lactam ring to generate a linear metabolite incapable of binding to PBPs, is the most prominent acquired mechanism for β-lactam resistance, notably in Gram-negative microbes. β-Lactamase enzymes can be mediated by the chromosome or easily transmitted by transposable elements. The most serious issues with β-lactamases include their extensive distribution throughout the microbial environment, their capacity to travel across vastly dissimilar organisms, their proclivity for swiftly inhibiting novel antibiotic drugs, and their development of resistance to β-lactamase inhibitors. Point mutations in various β-lactamases have arisen more recently, resulting in extended-spectrum β-lactamases (ESBLs) in Klebsiella pneumoniae that hydrolyze the most current cephalosporins. Extended-spectrum β-lactamases were the first plasmid-encoded β-lactamases with the ability to hydrolyze cephalosporins (ESBLs). These ESBL microorganisms are horizontally transferred by mobile genetic material from food, animals, or family members, and they cause more mortality than enteric bacilli that do not have these ESBLs (https://www.sciencedirect.com/science/article/pii/B9780323393072000333) (39). Only one intrinsic β-lactamase gene, *bla*_OXA-114_, from the class D family, has previously been discovered. However, as previously stated, this enzyme is a narrow-spectrum β-lactamase that likely plays a secondary role in the final β-lactam resistance profile of A. xylosoxidans. This is in line with the findings of multiple different forms of intrinsic β-lactamases (based on biochemical experiments): two cephalosporinases (40), two penicillinases (40–42), and an oxacillinase (43), which were described much earlier. At least five genes for β-lactamases have recently been discovered in the genome of A. xylosoxidans, and we believe that these genes are the primary determinants of this bacterium’s intrinsic β-lactam resistance (44). The primary focus of carrying out this experiment was to investigate the resistance against various antibiotics that our *A. mucicolens IA* strain may possess, either inherited or acquired from other bacteria. All categories of genes predicted in the historical strain *A. denitrificans* are considered essential and contribute to species’ survival in harsh situations (45).

### Conclusion.

Antibiotic resistance has spread widely as a result of a complex combination of parameters involved in survival adaptability and hence inheriting or gaining resistance genes. The overuse of antibiotics in clinical practice, which has resulted in bacteria developing newer resistances, is one key component in this adaptation. Furthermore, the epidemiology of antibiotic resistance in the environment remains unknown, making any predictions about the possibility of new antibiotic resistance spreading and emerging problematic. As a result, it is critical to figure out where resistance genes come from and whether they are passed down within a species or acquired from bacteria far away. Hence, the emergence of new resistance organisms is of clinical concern. When it comes to the clinical spectrum of infections by infrequent mechanisms, the significant opportunistic pathogen *A. mucicolens* has enhanced our knowledge. The isolated organism was cultured and subjected to antimicrobial susceptibility testing against standard drugs in the Vitek 2 system and also identified through a whole-genome sequencing approach. We tested the antibiotic resistance profile of the *A. mucicolens IA* strain and found out the various possible inherited as well as acquired regions that contribute toward our strain’s antibiotic resistance. In addition, we predicted the metabolic pathways encoded by the genes for proteins of the *A. mucicolens IA* strain. Hence, our research gives an overview of the *A. mucicolens IA* strain drug class resistance as well as information on acquired and inherited antibiotic resistance genes. With the use of comparative analysis for other strains of *A. mucicolens*, more research into the genes responsible for these drug resistance mechanisms can be done to gain a better understanding of the inherited and acquired genes.

## MATERIALS AND METHODS

### Bacterial strains and antimicrobial susceptibility tests.

This strain of *Achromobacter* was isolated from a sputum sample of a 47-year-old leukemia patient at a Baghdad teaching hospital suffering from persistent cough and nonresponding for multiple antibiotic drug combination therapy in the hemato-oncology department.

This sample was collected, *A*. *mucicolens* bacteria were cultured on MacConkey agar plates, and further obtained pure cultures were used for its identification, drug resistance tests, and whole-genome sequencing. The species was identified with biochemical tests using the Vitek 2 system (bioMérieux Vitek Systems Inc., Hazelwood, MO).

Antimicrobial susceptibility testing involved the MICs of various antibiotics reported for the *A. mucicolens* isolate from an automated microbiology identification system (Vitek 2). The antibiotic drugs included in the test were piperacillin-tazobactam, cefazolin, ceftazidime, ceftriaxone, cefepime, imipenem, amikacin, gentamicin, ciprofloxacin, levofloxacin, tigecycline, and trimethoprim-sulfamethoxazole. These MICs were then analyzed using both CLSI 2015 and EUCAST 2015 guidelines and classified as resistant, intermediate, or susceptible. We compared the susceptibility and agreement between the CLSI and EUCAST categorizations.

### Genome sequencing, assembly, and annotation.

The complete genome sequence was obtained by using sequencing-by-synthesis (SBS) technology, a next-generation sequencing (NGS) technology on the Illumina platform (Illumina Inc.) at Macrogen, South Korea. The sequencing library was prepared using the TruSeq DNA sample prep kit. DNA fragmentation was achieved by ultrasonication, and then adapter ligation and PCR enrichment were done. Paired-end reads were generated using a sequencing instrument, NovaSeq 6000. The whole-genome assembly was evaluated using FastQC v0.11.7 before and after trimming. Reads were trimmed (including adapter removal) using Trimmomatic v0.38. to discard sequences with per-base sequence quality score. For *de novo* assembly, default parameters without reference sequence were used to run Unicycler v0.4.7 and SPAdes v3.13.0. Only contigs with a size higher than 1,000 bp were kept. CONTIGuator 2.7.4 was used to obtain structural insight on the genomic sequence, giving a mapped contig file with reduced scaffolds under the reference sequence. The whole-genome sequence was annotated using the software Prokka 1.12 (46) to obtain gene details, coding regions, and protein information and also using RAST 2.0 (47–49).

### Bioinformatics.

The whole-genome sequence was analyzed with numerous bioinformatics tools. The annotation for gene details, coding regions, and protein information was retrieved using the software Prokka 1.12 and RAST 2.0, to obtain the information on numerous genes that contribute toward different antibiotic resistance and compare their presence to that in other strains of *A. mucicolens* (S. A. Al-Asadi, R. E. S. Al-Kahachi, W. M. A. Alwattar, J. Bootwala, and M. A. Sabbah, unpublished data).

Horizontally gene-transferred phage sequences and genomic islands were predicted by using Phaster (50, 51) (https://phaster.ca/) and IslandViewer 4 (52) online tools (www.pathogenomics.sfu.ca/islandviewer/) that combine the prediction results of three algorithms of genomic island identification: IslandPick (Langille et al., 2008) (31), SIGI-HMM (53), and IslandPath-DIMOB (54).

The tool antiSMASH 6.0 (55) (https://antismash.secondarymetabolites.org/) was used for the prediction of secondary metabolite biosynthesis gene clusters based on profile hidden Markov models of genes that are specific for certain types of gene clusters. The tool Tandem Repeats version 4.09 (56) (https://tandem.bu.edu/) was used for the prediction of tandem repeats to determine inherited genomes and the phenotypic variation.

The Clusters of Orthologous Groups (COGs) of proteins was used and understanding the classification of encoded proteins in the complete bacterial genome was performed using the COG database of NCBI.

### Search for drug resistance genes.

*Achromobacter* species are well known for exhibiting resistance to multiple drugs. In order to find out which antibiotics our strain *A. mucicolens IA* was resistant to, we used the Comprehensive Antibiotic Resistance Database (CARD) to test it. All the coding regions of genes were matched for the level of similarity to the genes in the Antibiotic Resistance Database (ARDB) associated with a certain type of resistance, which used BLAST hits to find drug resistance genes. Parameters of E value and percent identity were used to filter the significant hits. Antimicrobial resistance (AMR) genes, their products, and associated phenotypes were predicted using CARD with a BLASTN bit score cutoff parameter. AMR gene families and to which classes of drugs they show resistance mechanisms were also reported.

### Data availability.

The whole genome of the *A. mucicolens* strain was sequenced with next-generation sequencing on Illumina (NovaSeq 6000) and has been submitted to GenBank under the BioProject number PRJNA224116. The accession number is CP082965, and the version described in this study is CP082965. This *A. mucicolens* strain by subspecies classification is named the *IA* strain. The isolated bacterium was primarily reported as *A. denitrificans* by testing with the biochemical method of the Vitek 2 system, but the whole-genome sequence analysis and the sequence analysis of a single locus in the multilocus sequence typing (MLST) scheme, the *nrdA* 765-bp sequence, noted the isolated organism to be *A. mucicolens*. All *nrdA* sequences can be found at the PubMLST site at http://pubmlst.org/achromobacter (57).
